# Optimal Strategy for HIFU-Based Renal Sympathetic Denervation in Canines

**DOI:** 10.3389/fcvm.2021.739560

**Published:** 2021-10-14

**Authors:** Qingyao Liao, Fang Yang, Bo Xiong, Xiaoyu Zheng, Yue Wang, Jun Qian, Zhao Qiao, Jing Huang

**Affiliations:** ^1^Department of Cardiology, The Second Affiliated Hospital of Chongqing Medical University, Chongqing, China; ^2^Department of Clinical Medicine, Chongqing Medical and Pharmaceutical College, Chongqing, China; ^3^Ultrasonic Technology Center, Institute of Acoustics, Chinese Academy of Sciences, Beijing, China

**Keywords:** hypertension, renal sympathetic denervation, high intensity focused ultrasound, pre-clinical study, ultrasound

## Abstract

**Background:** The association between the treatment efficacy and safety of high-intensity focused ultrasound (HIFU)-based renal sympathetic denervation (RDN) and the acoustic energy dose applied has not been fully studied and may provide important understanding of the mechanism that led to failure of the WAVE IV trial. The objective of this study was to externally deliver different HIFU doses to canines for RDN treatment and to investigate the optimal energy dose for HIFU-based RDN.

**Methods:** Thirty canines were divided into five RDN groups according to dose of acoustic energy applied, and a sham control group that consisted of four canines was used for comparisons. All animals in the RDN groups underwent the RDN procedure with different acoustic energy doses, while in the sham control group, renal arteries were harvested without being subjected to acoustic energy delivery and were imaged using color Doppler flow imaging (CDFI). Blood pressure (BP) was recorded, and blood samples were collected before the RDN procedure and at 28 days after the RDN procedure. Histological examinations and measurement of renal tissue norepinephrine concentration were performed in all retrieved samples.

**Results:** Suppression of BP was significant in the 300 W (15.17/8.33 ± 1.47/1.21 mmHg), 250 W (14.67/9.33 ± 1.21/1.37 mmHg), and 200 W (13.17/9.17 ± 2.32/1.84 mmHg) groups. Semiquantitative histological assessment of periarterial nerves around the kidney revealed that target nerves in the 300 W (9.77 ± 0.63), 250 W (9.42 ± 0.67), and 200 W (9.58 ± 0.54) groups had the highest nerve injury scores, followed by the 150 W group (5.29 ± 0.62). Furthermore, decreased renal tissue norepinephrine concentration, together with decreased expression of tyrosine hydroxylase in the 300, 250, and 200 W groups demonstrated effective sympathetic depression following sufficient acoustic energy deposition. However, the renal artery injury score in the 300 W group (0.93 ± 0.13) was significantly higher than in the other groups (*p* < 0.001).

**Conclusion:** This study provides evidence that RDN effectiveness is based on the energy dose delivered and that 200–250 W is effective and safe in normal-sized canines.

## Introduction

Hypertension imposes a substantial burden on modern society and causes significant cardiovascular mortality and morbidity. Renal sympathetic denervation (RDN), which disrupts renal sympathetic nerves and diminishes sympathetic tone, has emerged as a promising device-based therapy for hypertension. Although intravascular RDN has recently achieved some success with either radiofrequency energy or ultrasound catheters (1–7), there remain limitations to the use of this technique, including restrictions on suitable candidate screening, risk of local endothelial damage, necessity for an interventional lab, and the invasive nature of the procedure. Therefore, new devices should be used to overcome the deficiencies of intravascular RDN.

High-intensity focused ultrasound (HIFU) can non-invasively deliver focused ultrasound energy to deep sites of the body without causing obvious damage to adjacent tissues. It has traditionally been used to treat uterus, kidney, and liver tumors (8–10). In addition, application of HIFU has been expanded to the area of RDN in recent years. Three studies (WAVE I, II, and III) (11) were conducted using the Kona Surround Sound System and have achieved encouraging outcomes. Because of the lack of a sham control group, which is believed to be mandatory at present, the results of the WAVE I–III trials are considered inconclusive. Therefore, the WAVE IV (12) study with a sham control group was carried out to verify data from the previous studies. However, similar decreases in systolic blood pressure (SBP) in the RDN and sham control groups were observed in the WAVE IV study, which led to this approach having a conflicting status. This raises the question as to whether acoustic energy applied externally causes effective RDN, as the encouraging outcomes of the WAVE I–III trials may have been the result of the placebo effect. However, other animal studies and clinical trials (13, 14) have suggested that this methodology can sufficiently ablate renal sympathetic nerves and reduce the magnitude of blood pressure (BP) to levels similar to those observed in studies using intravascular techniques. Therefore, careful refinement of the study protocols should be undertaken to overcome shortcomings that may have confounded previous results.

A prior study (13) demonstrated that inclusion of patients with uncontrolled hypertension at severe stages and large artery stiffness, and less stringent criteria for BP stabilization may have led to the disappointing outcomes of the WAVE IV study. Furthermore, as with the invasive RDN procedure, insufficient energy deposition at the nerves in non-invasive RDN procedures may lead to failure of nerve disruption and BP reduction, which is regarded as another important factor that led to failure of the WAVE IV study. In contrast, acoustic energy largely exceeds the threshold for disrupting renal sympathetic nerves and may cause damage to tissues on the acoustic pathway and those adjacent to the target area. Therefore, investigating the proper acoustic energy level to externally denervate renal sympathetic nerves effectively and safely is of great importance. The objective of this study was to externally deliver different levels of HIFU energy to canines for RDN treatment, and to compare the efficiency and safety between different energy levels to investigate the optimal energy dose for HIFU-based RDN.

## Materials and Methods

### High-Intensity Focused Ultrasound System for Renal Sympathetic Denervation

**Figure 1:**
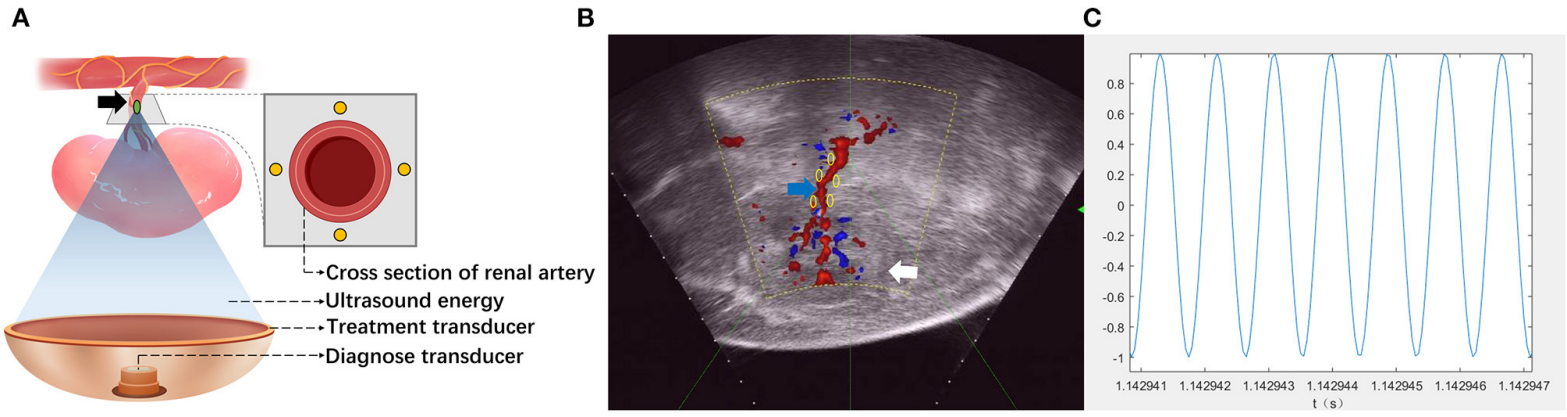
Schematic of high-intensity focused ultrasound (HIFU) for renal sympathetic denervation (RDN). **(A)** Illustrating the simultaneous treatment and imaging modules of the HIFU system. The target of the HIFU system (*green spot*) was set near the wall of the renal artery. Locations of the treatment site on the cross section of renal artery appear as *yellow spots*. *Black arrow*: renal artery. **(B)** The target of HIFU system (*green spot*) was targeted by three-dimensional movement of the treatment transducer guided by color Doppler flow imaging (CDFI). The locations of treatment sites on the longitudinal section of renal artery appear as *yellow spots*. *White arrows*, kidney; *blue arrow*, renal artery. **(C)** The waveform of the released ultrasound energy.

Focused acoustic energy was delivered using a HIFU system (Figure 1A) with a 130-mm spherically curved therapeutic transducer with a focal length of 128 mm. The operating frequency of the therapeutic transducer was approximately 1.12 MHz. The physical focal region detected with a needle-type hydrophone (NH0500, Precision Acoustic Co., Dorchester, Dorset, UK) was ellipsoid and had dimensions of 1.4 × 1.6 × 10 mm^3^. A diagnostic probe was aligned coaxially to the therapeutic transducer to locate target tissues. By adjusting the transducer in three dimensions, the focus could be moved 1 mm by 1 mm to target the renal artery.

### Animal Preparation

All canines used in this study were housed in individual cages in isolation from others. Room humidity and temperature were maintained at constant levels. All animals were over 5 years old and weighed ~12–15 kg. General anesthesia was induced with pentobarbital sodium (15 mg/kg) and an α_2−_receptor agonist (Su-Mian-Xin, 0.8 mg/kg). The trachea was cannulated, and the canines were ventilated mechanically with 2–3% isoflurane for maintained anesthesia. The right femoral was cannulated for invasive BP measurement using a PowerLab data acquisition system (AD Instrument, Australia). The left femoral vein was cannulated for intravenous access. The hairs on the backs of the animals were removed using a depilatory agent. Furthermore, the skin on the acoustic pathway was degreased with 75% alcohol and degassed with an aspirator. When the muscles of the animals were relaxed, the corneal reflex had disappeared, and BP was stable, we considered animals to have achieved a sufficient depth of anesthesia. Venous blood samples from all animals were preserved for plasma renal function examination before the RDN procedure.

All experiments performed in this study were approved by the Animal Ethics Committee of the Capital Medical University, China (AEEI-2021-060). The use and care of the animals were in compliance with the US National Institutes of Health Guide for Care and Use of Laboratory Animals.

### Experimental Protocols

Animals underwent the HIFU-based RDN procedure and were divided into five groups according to the dose of acoustic energy applied. Each group consisted of six animals. Additionally, a sham control group (*n* = 4) was employed for comparison. The backs of animals were immersed in a chamber that was filled with degassed water to provide an ideal environment between the animals and the acoustic transducer. Color Doppler flow imaging (CDFI) (Figure 1B) was used to obtain images of the long axis of the renal artery. With the guidance of CDFI, the focus of the HIFU system was set at the proximal, middle, and distal segments of the renal artery. At each segment, the focus was set at ~2 mm from the intima in the cranial, caudal, ventral, and dorsal directions. In the different groups, non-invasive sympathetic nerve denervation was performed on each target with a specific acoustic power (100, 150, 200, 250, and 300 W) and a duration of 3 s × 4 times. Figure 1C showed the waveform of the released ultrasound energy. A total of 12 emissions were delivered when each segment of the renal artery was visible under ultrasonographic view. When the RDN procedure on one side was completed, the same procedure was conducted on the other side of the animal. In the sham control group, only images of the renal artery were acquired without acoustic energy delivery. BP before and during surgery was recorded, and blood samples from all animals were collected for further analyses.

### Post-ablation Procedures

At 28 days after the RDN procedure, the invasive BP of all the animals was measured with the PowerLab data acquisition system, and bilateral renal artery CDFI images were obtained. Blood samples from all animals were preserved for post-ablation analyses. All animals were sacrificed by injection of 10% potassium chloride. The bilateral renal arteries, kidneys, as well as surrounding fat tissue were harvested for further analyses.

### Norepinephrine and Renal Function Examination

A total of 0.1–0.2 g of renal cortex tissue was obtained from the harvested kidneys for the measurement of renal tissue norepinephrine. After being frozen at −80°C, renal cortex tissue was homogenized in ice-cold 1% formic acid. The homogenate was then centrifuged at 13,000 rpm for 10 min. The supernatant was stored at −80°C prior to analysis. Norepinephrine concentration in renal tissue was measured via liquid chromatography mass spectrometry. Renal tissue norepinephrine concentration (ng/g) was calculated using the following formula: renal tissue norepinephrine concentration (ng/ml) × homogenate volume (ml)/renal tissue weight (g). The renal tissue norepinephrine concentrations of each RDN group were then compared with the sham control group. Norepinephrine data pertaining to each RDN group are presented as the percentage of norepinephrine reduction compared with the control group. Blood urea nitrogen (BUN) and serum creatinine (sCr) were measured using standard procedures before and 28 days of post-ablation.

### Histological and Immunohistochemical Evaluation

The harvested bilateral renal arteries, kidneys, and surrounding fat tissues were fixed in neutral formalin at 4°C. A total of six to eight sections from each renal artery and surrounding tissues were sectioned at 4- to 5-mm intervals, and were then routinely processed, paraffin embedded, and sliced for histological and immunohistochemical analyses. Hematoxylin and eosin staining was used for the evaluation of safety and efficacy of the RDN procedure. Histological assessment of the renal artery, adjacent nerves, and surrounding tissues in target regions of each segment was conducted using light microscopy (ICC50 HD, LEICA, Switzerland), and ordinal data were collected for multiple parameters, including endothelial cell loss, arterial medial injury (depth and circumference), inflammation, soft tissue injury, and necrosis. These parameters were semiquantified using a scoring system (15, 16) from 0–4 (e.g., none = 0, minimal = 1, mild = 2, moderate = 3, severe = 4). Table 2 describes typical histological changes reflecting injuries of the renal artery and adjacent nerves, as well as their classification and scoring. After the scoring was performed, various values were calculated and are presented in Table 3. Immunohistochemical staining was performed using tyrosine hydroxylase (1:300 dilution; PL Laboratories Inc., Port Moody, British Columbia, Canada) antibodies for the presence or absence of norepinephrine synthesis. The intensity of immunostaining was assessed according to a semiquantitative ordinal grading system from 0 to 3 (e.g., no reaction = 0, patchy/very weak reaction = 1, weak to moderate reaction = 2, strong reaction = 3) (15) (Table 2). Typically, a weak reaction or absence of tyrosine hydroxylase (TH) indirectly indicated degeneration of functional nerves and was associated with reduction of norepinephrine.

### Statistical Analysis

All data were analyzed using SPSS version 23.0 (IBM SPSS Statistics; IBM Corporation, Armonk, NY, USA). Results pertaining to continuous variables are presented as mean ± standard deviation. The Shapiro–Wilk test was used to statistically assess the normality of the data. Statistical comparisons between baseline and 28 days of follow-up were performed using the Wilcoxon signed-rank test for variables with skewed data distribution. The Kruskal–Wallis test was used for comparisons of mean BP reduction, renal function changes, renal tissue norepinephrine concentration, mean periarterial nerves, tissue injury score, and mean nerve immunostaining score between the different groups. All *p*-values were two-tailed, and differences with *p* < 0.05 were deemed statistically significant.

## Results

### BP Measurement

**Figure 2:**
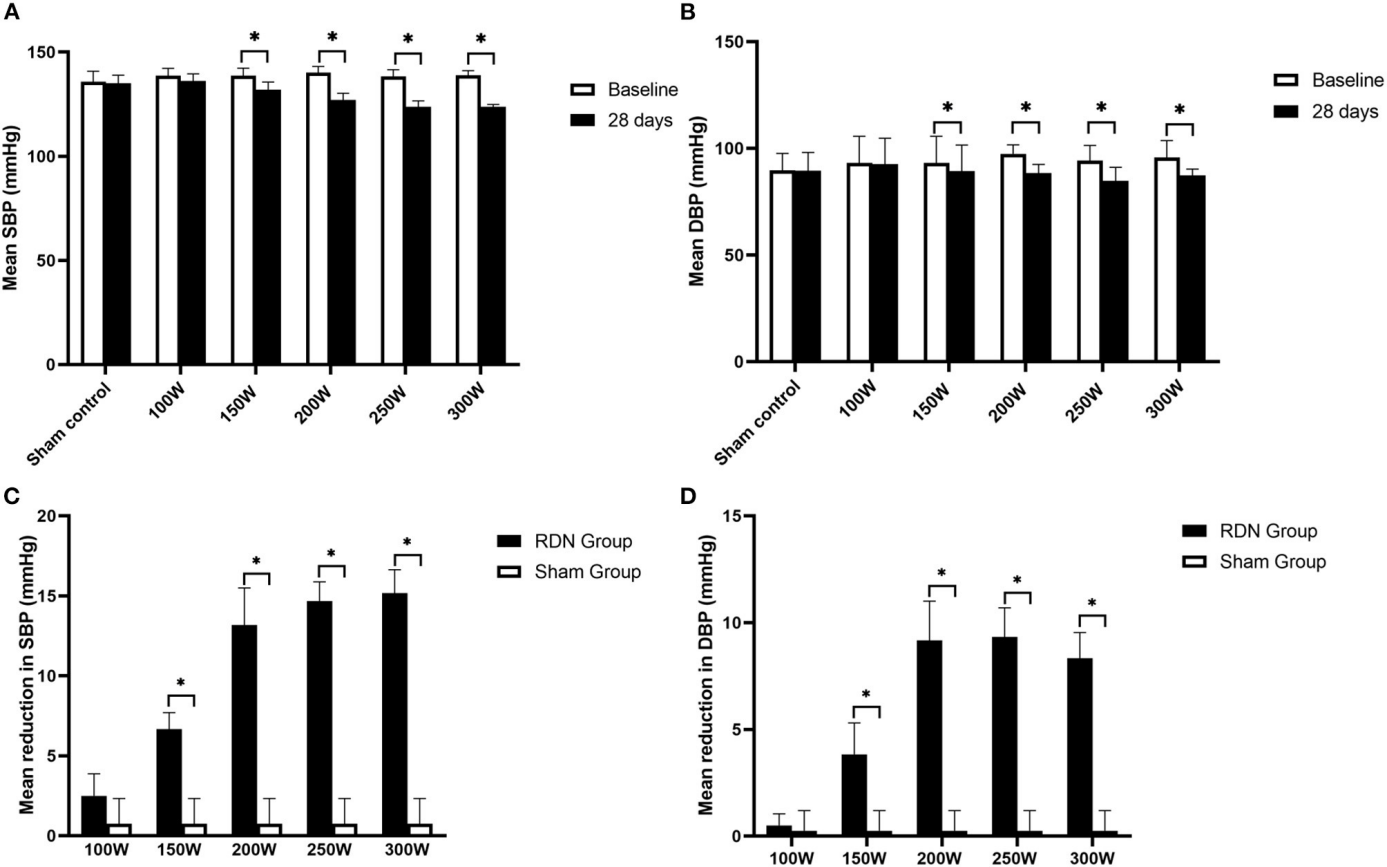
Mean SBP, DBP at baseline and day 28 after RDN. **(A, B)** Comparison of mean SBP, DBP between baseline and at day 28. **(C, D)** Mean reduction in SBP, DBP at day 28. SBP, systolic blood pressure; DBP, diastolic blood pressure. * < 0.05.

Comparison of BP at baseline and at day 28 is shown in Table 1 and Figure 2. The mean BP at baseline was not significantly different between the different groups. However, on day 28 after the RDN procedure, the mean SBP in the 300 W (123.67 ± 1.21 mmHg, *p* = 0.027), 250 W (123.67 ± 2.94 mmHg, *p* = 0.027), 200 W (127.00 ± 3.23 mmHg, *p* = 0.026), and 150 W (132.00 ± 3.69 mmHg, *p* = 0.026) groups and mean diastolic blood pressure (DBP) in the 300 W (87.33 ± 2.94 mmHg, *p* 0.027), 250 W (84.83 ± 6.27 mmHg, *p* = 0.026), 200 W (88.33 ± 4.13 mmHg, *p* = 0.026), and 150 W (89.33 ± 12.24 mmHg, *p* = 0.026) groups were statistically decreased compared with baseline. Furthermore, as shown in Figure 2, BP reduction was greatest in the 300, 250, and 200 W groups (−15.17/−8.33 ± 1.47/1.21, −14.67/−9.33 ± 1.21/1.37, and −13.17/9.17 ± 2.32/1.84 mmHg, respectively) and showed no statistical differences (SBP: *p* = 0.513, DBP: *p* = 0.472), followed by the 150 W group (−6.67/−3.83 ± 1.03/1.47 mmHg), and the lowest reduction in BP was observed in the 100 W group, which showed no significant difference with the sham control group (−2.50/0.50 ± 1.38/0.55 vs. 0.75/0.25 ± 1.58/0.95 mmHg, SBP: *p* = 0.097; DBP: *p* = 0.724).

**Table 1:** Mean blood pressure (BP) in renal sympathetic denervation (RDN) and sham control groups at baseline and day 28.

	**RDN Group (*****n*** **=** **30)**						
**Variable**	**300 W Group**	**250 W Group**	**200 W Group**	**150 W Group**	**100 W Group**	**Sham control group**	***p*-Value**
	**(*n* = 6)**	**(*n* = 6)**	**(*n* = 6)**	**(*n* = 6)**	**(*n* = 6)**	**(*n* = 4)**	
Baseline SBP (mmHg)	138.83 ± 2.31	138.33 ± 3.26	140.17 ± 2.93	138.67 ± 3.61	138.67 ± 3.56	135.75 ± 5.08	0.645
Baseline DBP (mmHg)	95.67 ± 1.97	94.17 ± 7.19	97.50 ± 4.18	93.67 ± 12.45	93.17 ± 12.45	89.75 ± 7.93	0.627
28-Days SBP (mmHg)	123.67 ± 1.21	123.67 ± 2.94	127.00 ± 3.23	132.00 ± 3.69	136.17 ± 3.37	135.00 ± 3.91	<0.001
28-Days DBP (mmHg)	87.33 ± 2.94	84.83 ± 6.27	88.33 ± 4.13	89.33 ± 12.24	92.67 ± 12.04	89.50 ± 8.58	0.247

*Values are expressed as mean ± SD. SBP, systolic blood pressure; DBP, diastolic blood pressure*.

*Continuous variables were analyzed using Kruskal–Wallis test*.

**Table 2:** Semiquantitative Scoring System for evaluating the injury of renal artery and adjacent nerves.

**Histological assessment of renal artery and adjacent nerves**					
Injury classification	No injury	Minimal injury	Mild injury	Moderate injury	Severe injury
Scoring	0	1	2	3	4
Histological injury found in nerves	No abnormal findings	Minimal vacuolization	Mild vacuolization; mild pyknotic nuclei; rare digestion chambers	Severe vacuolization; moderate pyknotic nuclei; frequent digestion chambers;	Effacement of nerve architecture; necrosis; retraction
Histological injury found in arteries	Medial change depth 0% of media thickness	Medial change depth <25% of media thickness	Medial change depth 25– <50% of media thickness	Medial change depth 50– <75% of media thickness	Medial change depth >75% of media thickness
**Immunohistochemistry assessment of nerves adjacent to renal artery**					
Reaction to immunostaining	No reaction	Patchy/very weak reaction	weak to moderate reaction	Strong reaction	
Scoring	0	1	2	3	
Intensity and distribution of immunostaining	No staining for TH	Patchy/very weak staining for TH	Weak to moderate staining for TH	Strong staining for TH	

**Table 3:** Values calculated from the semiquantitative scoring systems.

**Values**	**Definition**
Sum of the nerve injury/immunostaining score and sum of the renal artery injury score per animal	Calculated by summing up all the treatment sites' scores of all the renal artery segments (typically 6–8 segments). This was done for the vascular injury and the nerve injury separately.
Mean nerve injury/immunostaining score and mean renal artery injury score	Calculated by dividing the sum of the vascular/nerve injury score, by the number of arterial segments in the given animal.

### Renal Tissue Norepinephrine Concentration Examination

**Figure 3:**
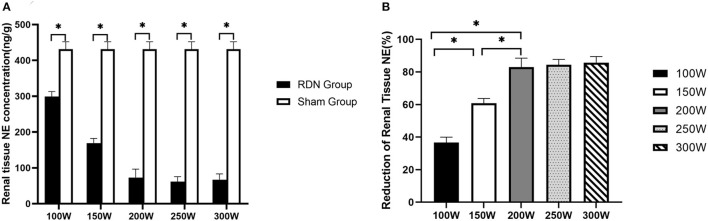
Renal tissue NE concentration at day 28. **(A)** Comparison of mean renal tissue NE concentration between RDN groups and sham control group at day 28. **(B)** Reduction in renal tissue NE in RDN groups at day 28. NE, norepinephrine. * < 0.05.

As shown in Figure 3, on day 28 after the RDN procedure, renal tissue norepinephrine concentration was significantly lower in the RDN groups compared with the sham control group (431.40 ± 20.73 ng/g, *p* = 0.011). When comparisons were made between the different RDN groups, no significant differences were observed between the 300 W (67.31 ± 16.00 ng/g), 250 W (61.46 ± 14.12 ng/g), and 200 W groups (73.20 ± 23.41 ng/g) (*p* = 0.587). However, renal tissue norepinephrine concentration was notably lower in the 300, 250, and 200 W groups than in the 150 W (169.25 ± 12.86 ng/g, *p* = 0.004) and 100 W groups (299.06 ± 14.15 ng/g, *p* = 0.004). Additionally, compared with the 100 W group, renal tissue norepinephrine concentration was significantly lower in the 150 W group (*p* < 0.05). Moreover, with the increase in acoustic energy dose applied in the RDN procedure, reduction of renal tissue norepinephrine compared with the sham control group was increased (30.66%, 60.76%, 83.03%, 84.39%, 85.75% from 100 to 300 W, respectively) (Figure 3), which indicated that more sympathetic activity was reduced by HIFU ablation at higher acoustic energy levels.

### Histological and Immunohistochemical Evaluation

**Figure 4:**
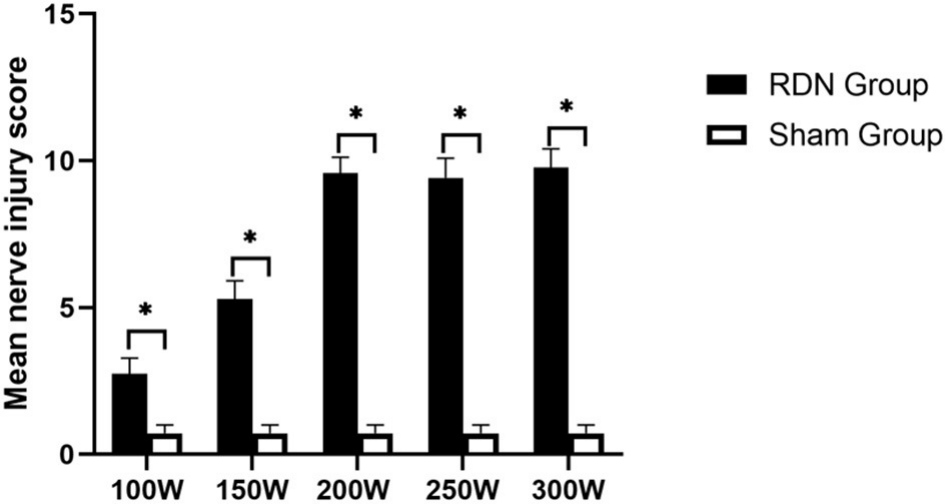
Semiquantitative histological assessment of periarterial nerves at day 28; * < 0.05.

**Figure 5:**
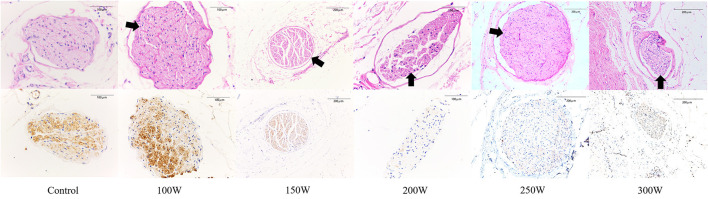
Representative histological and immunohistochemical images of nerves in different groups at day 28. (*First row*) Representative images of nerves with hematoxylin and eosin (H&E) stain. (*Second row*) Corresponding images of nerves stained by anti-tyrosine hydroxylase (TH) enzyme. *Black arrow* indicates the vacuolization and pyknotic nuclei in nerves.

As shown in Table 4 and Figure 4, at 28 days of follow-up, the mean nerve injury score in the 300 W (9.77 ± 0.63, *p* = 0.011), 250 W (9.42 ± 0.67, *p* = 0.01), 200 W (9.58 ± 0.54, *p* = 0.011), 150 W (5.29 ± 0.62, *p* = 0.011), and 100 W (2.75 ± 0.53, *p* = 0.011) groups was statistically higher when compared with the sham control group (0.72 ± 0.28). Besides, in the RDN groups, mean nerve injury scores in the 300, 250, and 200 W groups showed no significant differences (*p* = 0.616) and were significantly higher than those in the 150 W (*p* = 0.004) and 100 W (*p* < 0.001) groups. Furthermore, the mean nerve injury score was significantly greater in the 150 W group compared with the 100 W group (*p* = 0.004). Regarding mean nerve injury score calculated according to type of change (e.g., degeneration, necrosis, chronic) and magnitude (e.g., minimal, mild, moderate, severe), the higher nerve injury scores in the 300, 250, and 200 W groups suggested nerve damage was severe in these groups compared with others. Representative images of renal nerve injury are shown in Figure 5. Moderate to severe injury of nerves was observed in the 300, 250, and 200 W groups, and was characterized by vacuolization and pyknotic nuclei in target nerves, while perineuronal tissue was inflamed with infiltration of mononuclear inflammatory cells. In the 150 W group, affected nerves showed mild to moderate changes including mild vacuolization with rare pyknotic nuclei. In the 100 W group, however, injury of nerves in the target area was considered minimal, as perineural inflammation was rare, and endoneural damage was limited. In the sham group, histological findings remained unchanged.

**Table 4:** Comparison of mean nerve and renal artery injury score between groups at day 28.

	**RDN Group (*****n*** **=** **30)**						
**Variable**	**300 W Group**	**250 W Group**	**200 W Group**	**150 W Group**	**100 W Group**	**Sham control group**	***p*-Value**
	**(*n* = 6)**	**(*n* = 6)**	**(*n* = 6)**	**(*n* = 6)**	**(*n* = 6)**	**(*n* = 4)**	
Mean nerve injury score	9.77 ± 0.63	9.42 ± 0.67	9.58 ± 0.54	5.29 ± 0.62	2.75 ± 0.53	0.72 ± 0.28	<0.001
Mean renal artery injury score	0.93 ± 0.13	0	0	0	0	0	<0.001

*Values are expressed as mean ± SD. Kruskal–Wallis test was used*.

**Figure 6:**
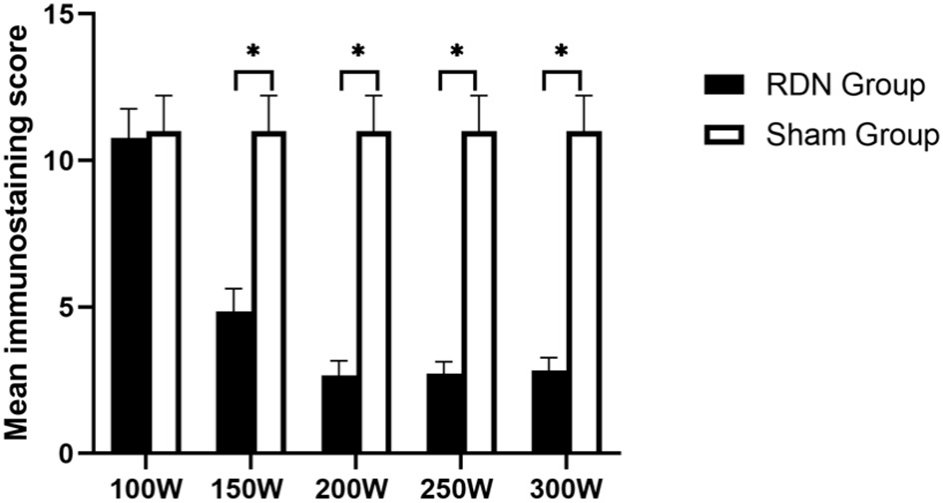
Semiquantitative immunohistochemical evaluation of periarterial nerves at day 28. * < 0.05.

Representative images of nerves after immunostaining are shown in Figure 5. Figure 6 shows the mean nerve immunostaining score in the different groups. When compared with the sham control group (11.00 ± 1.22), the mean nerve immunostaining score in the 300 W (2.83 ± 0.44, *p* = 0.011), 250 W (2.73 ± 0.40, *p* = 0.011), 200 W (2.67 ± 0.49, *p* = 0.011), and 150 W (4.85 ± 0.57, *p* = 0.010) groups was significantly lower, which represented an effective treatment effect in these groups. However, in the 100 W (10.77 ± 0.99) group, the reaction of TH was strong, and the mean nerve immunostaining score did not differ from that in the sham control group (*p* = 0.454), which demonstrated that target nerves in the 100 W group received insufficient acoustic energy deposition. Besides, when comparisons were made between the different RDN groups, most target nerves in the 300, 250, and 200 W groups showed weak or no reaction to TH, which represented a stronger treatment effect, and the mean nerve immunostaining scores were significantly lower compared with the 150 W (*p* = 0.004) and 100 W (10.77 ± 0.99 *p* = 0.01) groups. Moreover, compared with the 100 W group, the mean nerve immunostaining score in the 150 W group (*p* = 0.004) was significantly lower, which demonstrated that a portion of target nerves in the 150 W group received sufficient acoustic energy deposition and showed a weak reaction to TH.

### Safety Analysis

**Figure 7:**
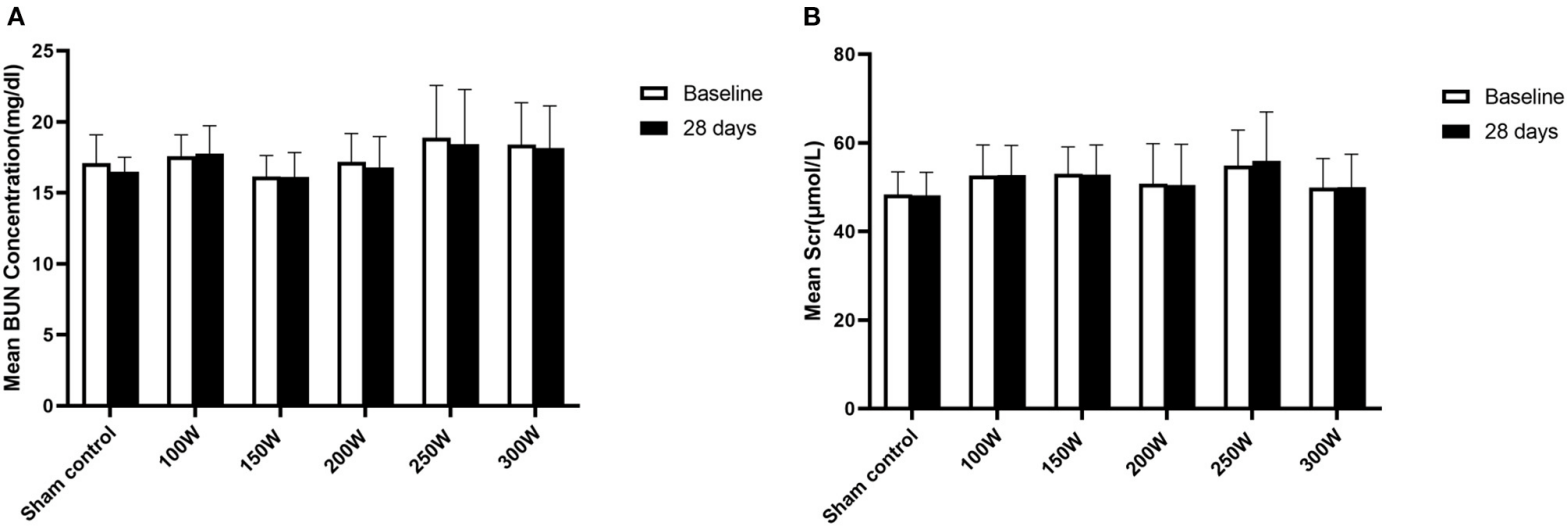
Renal function at day 28. **(A, B)** Presented changes in BUN and Scr at day 28, respectively.

**Figure 8:**
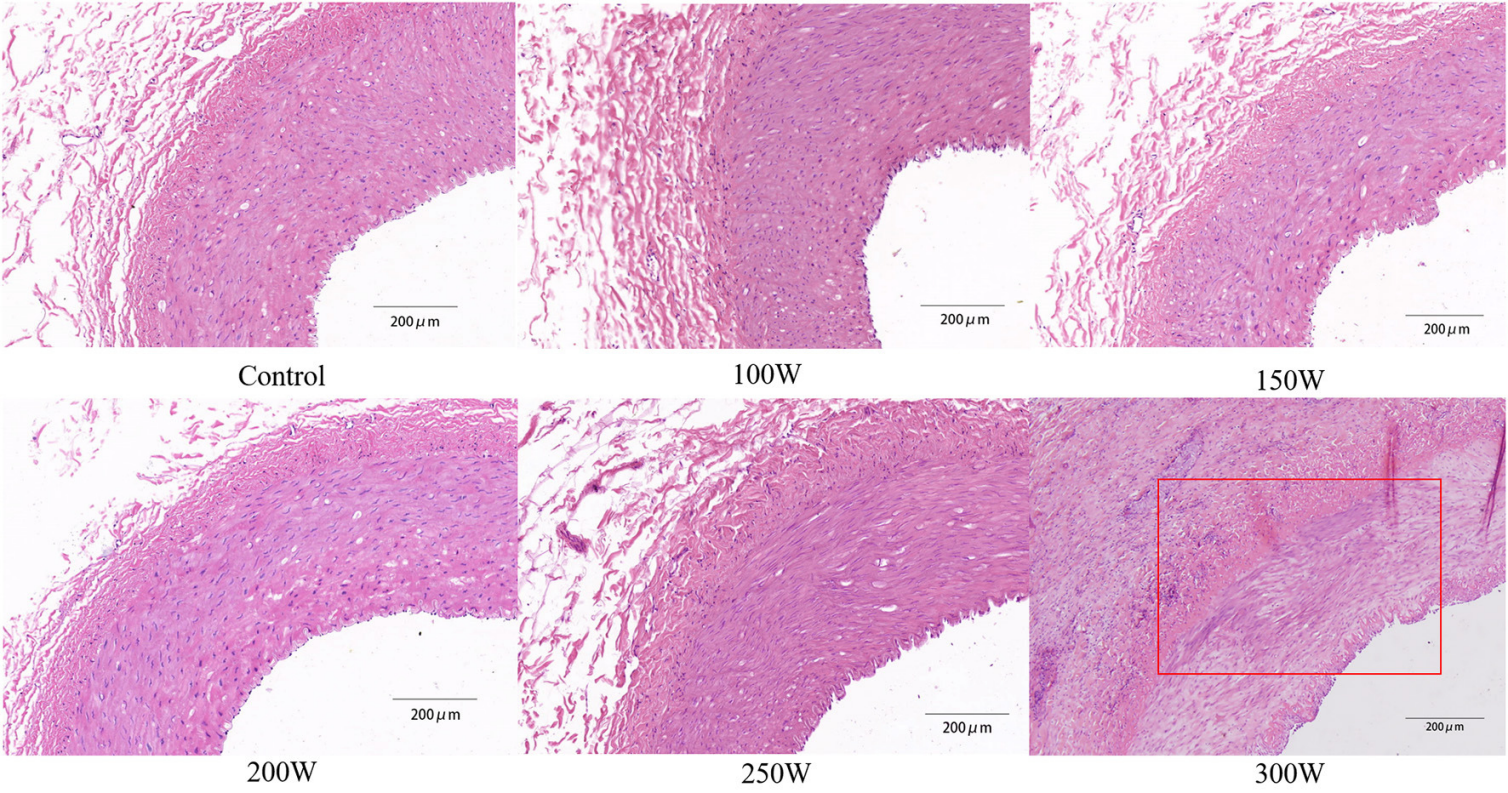
Representative histological images of renal artery in different groups. *Red rectangle* exhibits renal vascular muscle cell loss with proteoglycan replacement, which was the result of the thermal effect of HIFU energy.

All animals survived the RDN procedure and until the end of the observation period. The main vital signs of all animals were normal and stable until they were sacrificed. Gross assessment of tissues on the acoustic pathway revealed rare significant injury, including of the skin, liver, spleen, as well as tissues adjacent to target regions. No significant differences were observed in the BUN or sCr between baseline and 28 days of follow-up in either the RDN groups or sham control group (Figure 7). CDFI at 28 days after the RDN procedure showed normal renal arteries without signs of stenosis. Gross observation of the renovasculature revealed no obvious damage. Renal arteries harvested from all animals were smooth and exhibited an intact vascular wall without bleeding spots and ulcers. Histological examination revealed intact vascular structure with intact endothelium. However, at 28 days post-ablation in the 300 W group, renal vascular smooth muscle was observed with mild renal artery damage, which presented as muscle cell loss with proteoglycan replacement. Representative images of vessel walls are shown in Figure 8. Furthermore, as shown in Table 4, mean renal artery injury score in the 300 W group (0.93 ± 0.13) was significantly different from that in the other groups (*p* < 0.001), which demonstrated vascular risk in the higher-energy group.

## Discussion

In the present study, different levels of acoustic energy were externally delivered to the sympathetic nerves adjacent to the renal arteries of canines. Suppression of BP was significant in the 300, 250, and 200 W groups. Moreover, semiquantitative histological assessment of periarterial nerves around the renal arteries showed that nerves in the 300, 250, and 200 W groups had the highest nerve injury scores and exhibited more severe damage, followed by the 150 W group. Furthermore, decreased renal tissue norepinephrine concentration, together with the lower expression of TH in the 300, 250, and 200 W groups demonstrated effective sympathetic depression following sufficient deposition of acoustic energy. However, renal arteries in the 300 W group showed mild damage and had an artery injury score that was notably higher than in the other groups.

Over the past decades, intravascular RDN has achieved some success with different forms of energy. Although the outcome of the SYMPLICITY HTN-3 trial (17) was disappointing, a second-generation radiofrequency ablation system (Symplicity Spyral, Medtronic) was subsequently employed and achieved positive results in two clinical trials (SPYRAL HTN-OFF MED and SPYRAL HTN-ON MED) (3, 4). Furthermore, intravascular ultrasound-based RDN ablation systems (Paradise ultrasound system; ReCor Medical, Palo Alto, CA, USA) successfully suppressed BP in hypertensive patients in the RADIANCE-HTN SOLO and RADIANCE-HTN TRIO clinical trials (6, 7). These encouraging outcomes make intravascular RDN a promising method in hypertension treatment. However, there remain limitations with RDN when it is applied invasively. First, because the energy delivered from the intravascular catheter to perivascular nerves must traverse the renal artery, more energy is deposited on the arterial wall adjacent to the catheter than on the outer adventitial layer, resulting in injury to the endothelium. Moreover, energy attenuation on the arterial vessel wall may lead to insufficient energy deposition in the target region, resulting in incomplete disruption of sympathetic nerves, which is believed to be one of the main factors that led to failure of the HTN-3 trial (17). Additionally, not all patients are suitable candidates for intravascular RDN because there are specific anatomic requirements to be eligible for the procedure, such as renal arteries with a certain diameter and length for catheter insertion (18). Furthermore, catheter-based devices are invasive and require the use of fluoroscopy and contrast agents, which increase risks and complications for patients (19).

Because it can be performed externally and non-invasively, HIFU-based RDN can overlook the anatomic restrictions of arteries described above and is believed to be safer, rendering patients less susceptible to complications that occur with intravascular RDN. Over the past years, HIFU-based RDN has undergone extensive investigation and has achieved some success. Wang et al. (14) investigated the effectiveness of HIFU in sympathetic inhibition and lowering of BP in normotensive canines and achieved encouraging results. Furthermore, in a series of experiments involving 225 swine (20), the application of external acoustic energy was found to sufficiently ablate nerves around the renal artery. Furthermore, the WAVE I, II, and III clinical trials (11) carried out with the Kona Medical Surround Sound System have largely reduced office and ambulatory BP in patients with resistant hypertension. These encouraging results make HIFU-based RDN a promising antihypertensive technique. However, failure of the WAVE IV trial (12) led researchers to question the efficacy of RDN conducted via this non-invasive manner. Nonetheless, evidence provided by previous studies demonstrated sufficient sympathetic depression in response to externally delivered focused ultrasound and the magnitude of the BP response in clinical trials conducted with either the Medical Surround Sound™ system (Kona Medical, Inc., Bellevue, WA, USA) (8) or Model-JC HIFU tumor therapeutic system (Chongqing Haifu Technology Co. Ltd., Chongqing, China) (9) was similar to that observed with invasive procedures. Therefore, failure of the WAVE IV trial was believed to be influenced by certain confounding factors.

A previous study (13) demonstrated that inclusion of patients with severe uncontrolled hypertension and large artery stiffness, as well as less stringent criteria for BP stabilization may have partially hampered the interpretation of results in the WAVE IV trial. However, because of a lack of immediate confirmation of complete denervation of periarterial sympathetic nerves, insufficient ultrasonic energy deposition may have been another crucial factor that led to failure of the WAVE IV trial. As demonstrated in our study, from 100 to 200 W, BP suppression and renal sympathetic nerve damage were increased with increase in acoustic energy, and there were no further significant changes when acoustic energy was increased beyond 200 W, which suggests that the therapeutic efficacy of RDN is associated with an acoustic energy level within a specific range. Insufficiently focused ultrasound delivery can result in inadequate temperature elevation at the target region, which can lead to failure of complete sympathetic nerve disruption. Previous studies (18, 21, 22) highlighted the role of insufficient ablation on the variability of BP response observed following RDN. Furthermore, Sakura et al. (23) reported sympathetic nerve regeneration at 180 days after the RDN procedure with radiofrequency energy, which may be partially explained by insufficient energy delivery. However, as ultrasound energy increases, the temperature at the focal spot reaches that of the threshold for nerve necrosis, resulting in the treatment effect of RDN reaching a plateau. Therefore, to conduct effective non-invasive RDN procedures, an effective denervation energy threshold should be determined.

Another important factor attributed to insufficient energy deposition at the target nerve region may be distortions of the ultrasound beam induced by the overlying tissue layers. Because the acoustic energy is delivered externally, skin and fascia on the acoustic pathway may partially reflect or absorb some of the acoustic energy, resulting in insufficient energy deposition. As described by Freyhardt et al. (24), following the RDN procedure in pigs, magnetic resonance thermometry detected abnormal temperature elevation of tissues on the dorsal perirenal area of the target region, which demonstrated energy absorption on the beam path. The effective acoustic intensity and exposure time used in the present study were similar to those of the WAVE I–IV trials. However, because the distance from the skin to the bifurcation of the renal artery in humans is significantly longer than that in canines, more ultrasound energy is absorbed and reflected on the acoustic pathway when HIFU-based RDN is conducted in humans. Therefore, the acoustic energy deposited at the focal target may not be sufficient to completely denervate the sympathetic nerve adjacent to the renal artery, which leads to failure of RDN. Furthermore, Wang et al. (14) and Rong et al. (13) conducted HIFU-based RDN in canines and humans, respectively, via the same Model-JC HIFU tumor therapeutic system and achieved encouraging outcomes. Although the acoustic energy doses used in their studies were the same, the exposure times were significantly different (12 vs. 50 s), resulting in different cumulative acoustic energy. Therefore, when determining acoustic energy for RDN treatment, specific details of the acoustic window should be incorporated so as to achieve a successful RDN procedure. Although not included in the present study, calculating acoustic energy for renal denervation treatment by establishing models that incorporate all tissue types on the beam pathway should be carried out in the future.

While the 200 and 250 W RDN groups exhibited sufficient antihypertensive effects without adverse outcomes, histological evaluation of the renal artery in the 300 W group showed minimal to mild vascular wall injury and a significantly higher mean renal artery injury score than any other group. This phenomenon may be explained by the difference in sensitivity of vessels and nerves to acoustic energy. At lower acoustic energy levels, because the nerve fascicle consists of a multilayer lipid membrane structure, ultrasound can be reflected repeatedly, which contributes to acoustic energy deposition within the nerve fascicle and results in nerve susceptibility to ultrasound-mediated injury (25). Previous studies (26, 27) demonstrated that nerves can be selectively blocked at a relatively lower acoustic energy level, which suggests that nerves are more susceptive to ultrasound than other tissues. In contrast, because the ultrasound beam is delivered externally, there is no catheter inserted in the renal artery to block blood flow within the artery. Therefore, the rapidly circulating blood stream can cool the artery and further avoid injury to the vessel wall. However, when acoustic energy deposited to the target area is increasing, the thermal effect of focused ultrasound on the renal artery may be inevitable, resulting in obvious pathological changes to the vessel wall.

The present study had several limitations. First, the use of anesthesia in the animals may have affected the accuracy of BP measurements. Therefore, we took measures to overcome the influence of anesthesia, such as repeatedly assessing anesthetic depth and BP. Furthermore, our study was conducted in normotensive canines, and the features of arteries and acoustic pathway of canines may be different from hypertensive patients. Therefore, the results of our study may not necessarily apply to hypertensive humans. Furthermore, because nerve regeneration may occur after RDN procedures, a long-term follow-up study is necessary to fully evaluate the efficacy and safety of the HIFU strategy. Finally, because weight was not significantly different between experimental animals, we failed to detect an association between the distance of the beam path and the effect of the RDN procedure. Therefore, the loss of focused ultrasound on the pathway must be quantitatively investigated in the future.

In conclusion, our study demonstrated that the therapeutic efficacy of HIFU-based RDN is associated with acoustic energy levels within a specific range, which then reached a treatment plateau where the HIFU intensity was beyond the threshold. However, with the application of continuously increasing ultrasound energy, tissues adjacent to the target region may undergo damage, resulting in unnecessary side effects. These results provide evidence that the effectiveness of RDN is based on the dose of energy delivered, and that 200–250 W is effective and safe in normal-sized canines.

## Data Availability Statement

The raw data supporting the conclusions of this article will be made available by the authors, without undue reservation.

## Ethics Statement

The animal study was reviewed and approved by Animal Ethic Committee of the Capital Medical University, China.

## Author Contributions

QL, FY, and BX contributed to the study design, experiment performance, tissue harvest, analysis, and drafted and revised the manuscript. XZ contributed to the design and acquisition of data. YW was involved in the engineering support. JQ contributed to the acquisition of data. ZQ contributed to the statistical analysis. JH contributed to the design and critically revised the manuscript. All authors contributed to the article and approved the submitted version.

## Funding

This work was funded by the National Natural Science Foundation of China (No. 81900361).

## Conflict of Interest

The authors declare that the research was conducted in the absence of any commercial or financial relationships that could be construed as a potential conflict of interest.

## Publisher's Note

All claims expressed in this article are solely those of the authors and do not necessarily represent those of their affiliated organizations, or those of the publisher, the editors and the reviewers. Any product that may be evaluated in this article, or claim that may be made by its manufacturer, is not guaranteed or endorsed by the publisher.
